# Rationale, design, and characteristics of the multimedia family planning campaign for a small, happy, and prosperous family in Ethiopia (SHaPE)

**DOI:** 10.1186/s12889-018-5799-5

**Published:** 2018-07-11

**Authors:** Hye-Jin Paek, Ho Kim, Youngtae Cho, Wonsik Hong, Woorim Ko, Haejin Choi, Youngok Youn, Yunhee Choi, Gizachew Balew, Youngah Doh

**Affiliations:** 1 Department of Advertising & Public Relations, Hanyang University, 55 Hanyangdeahak-ro, Sangnok-gu, Ansan, Gyeonggi-do South Korea; 2 Graduate School of Public Health, Seoul National University, 1 Gwanak-ro, Gwanak-Gu, Seoul, South Korea; 3 Department of Liberal Arts College of Liberal Arts and Sciences, Dongduk Women’s University, Wharang-ro, 13 Gil, 60 Sungbook-Gu, Seoul, South Korea; 4 Korea Population, Health and Welfare Association, 20 Beodeunaru-ro 14ga-gil, Yeongdeungpo-gu, Seoul, South Korea; 5 EngenderHealth Ethiopia Office, Djibouti Avenue, Addis Ababa, Ethiopia; 6 Ethiopia Office, Korean International Cooperation Agency (KOICA), Addis Ababa, Ethiopia

**Keywords:** Family planning, Ethiopia, Multimedia campaign, Entertainment-education, Evaluation

## Abstract

**Background:**

Ethiopia, the second most populous country in Africa, has a total fertility rate of 4.6, a decrease from 5.5 in 2000. However, only 35.3% of women in the reproductive age group use modern family planning (FP) methods, and the 22.3% of them who have an unmet need for family planning is among the highest rates in sub-Saharan African countries. The Small, Happy, and Prosperous family in Ethiopia (SHaPE) is one of the country’s first comprehensive multimedia family planning campaigns. Its purpose is to increase FP-related knowledge, attitude, and practice of Ethiopians, particularly women of reproductive age.

**Methods/Design:**

The SHaPE campaign has multiple components: (1) a nationwide representative survey, which serves as formative research to identify region-specific and culture-appropriate media, messages, and barriers and determinants of family planning; (2) a multimedia communication campaign intervention, including radio dramas and other interpersonal, community-level, and mass media channels; and (3) campaign evaluation, including pre-, process-, and post-evaluation research using both quantitative and qualitative methodologies. The main target population for SHaPE is reproductive age women and men in three regions: Amhara, Oromia, and Somali. These regions take up about 66.6% of the entire country and have distinct ethnicities, cultures, and languages.

**Discussion:**

SHaPE contributes to existing family planning research and intervention because it is theory- and evidence-based, and it employs integrated marketing communications and entertainment-education approaches with key messages that are tailored to audiences within unique cultures. But even within a country, a nationwide campaign with uniform messages is neither possible nor desirable due to different cultures, norms, and languages across regions. Last, media campaigns in developing and underdeveloped countries require constant monitoring of political situations.

## Background

Despite global efforts to reduce maternal deaths, in 2015 two thirds of women (303,000) between the ages of 15 and 49 still died from causes related to pregnancy and childbirth, and about two thirds of the deaths (201000) occurred in Sub-Saharan Africa [1]. For reducing maternal death and other adverse health effects, family planning remains a highly cost-effective precautionary method [2]. Here, family planning (FP) refers to the ability of individuals and couples to anticipate and attain their desired number of children, as well as the spacing and timing of their births [3, 4]. Facilitation of family planning in countries with high birth rates could potentially avert 32% of all maternal deaths and nearly 10% of childhood deaths. In addition, family planning could contribute not only to women’s education and empowerment but to long-term improvements in economic development and public health [5].

As of 2017, Ethiopia, located in the Horn of Africa, is the continent’s second most populous country, with an estimated total population of over 100 million [6]. It has one of the fastest growing economies in the world [7]. However, it is also one of the world’s poorest nations, with a per capita GDP of 511 USD [8], and 83.8% of the population lives in rural areas. In 2017, its Human Development Index -- a composite index of life expectancy, education, and per capita income indicators -- was ranked 174 out of 188 countries, and its literacy rate was 49% [9]. Total fertility rate has decreased from 5.5 children per woman in 2000 to 4.6 within the last sixteen years [10]. However, only 35.3% of women in the reproductive age group have used modern family planning methods, and the unmet need for family planning is 22.3%, among the highest rate in sub-Sharan African countries [10, 11]. This unmet need for family planning, defined as “the condition of wanting to avoid or postpone childbearing but not using any method of contraception” [12], can be met through a strong family planning program that addresses people’s FP-related knowledge, beliefs, attitudes, and behaviors.

Due to its importance as a public health issue, family planning research in Ethiopia has been relatively well documented [11, 13–20]. Studies have found that women’s knowledge and habits of contraceptive use are generally low, and that family planning practice is determined by many socio-demographic factors such as women’s social status, women’s education, women’s age at marriage, and gender norms [13, 15, 21, 22]. Knowledge, subjective norms, and interpersonal communication with spouses were found to be significantly related to women’s willingness to use FP methods [11]. These findings imply the need for addressing multiple FP determinants at intrapersonal, interpersonal, and probably also community and cultural levels.

While previous research has attempted to identify determinants of FP, efforts to implement and assess intervention programs -- such as a national-level media campaign for improving knowledge, attitudes, and practices of family planning -- have been scarce. One intervention program provided FP services, and its evaluation research found that the intervention had positive impact on FP behaviors among both married women and their husbands [23]. Although there is still a vast knowledge gap regarding FP, recent statistics [10] show that the most common sources of family planning messages are community events (37%), followed by radio (34%). Taking these media environments into account, a multi-media campaign approach is required to fill in FP knowledge gaps and satisfy unmet FP needs.

## Methods/Design

### What is SHaPE?

The Small, Happy, and Prosperous Family in Ethiopia or SHaPE is one of the country’s first comprehensive multimedia campaigns. It has nationwide coverage and combines radio dramas with other forms of interpersonal, community-level, and mass media. Its purpose is to increase knowledge, positive evaluations, and practice of FP among Ethiopian people, particularly women of reproductive age. Family planning can be achieved through a variety of forms, such as contraceptive use to prevent unwanted pregnancy, childbirth spacing, delaying marriage and childbirth, giving women access to higher education, and empowering women. While programs focusing on contraceptive use may have a direct impact on family planning and reduce the fertility rate in the long term, they may be socio-culturally inappropriate and normatively unacceptable in communities where people are very religious, and where a majority of people live in rural areas and need more human labor for farming. Indeed, culture and norms vary greatly across regions in Ethiopia, and this variety calls for culture-specific tailored messages that coincide with different communities’ norms and cultures.

**Fig. 1:**
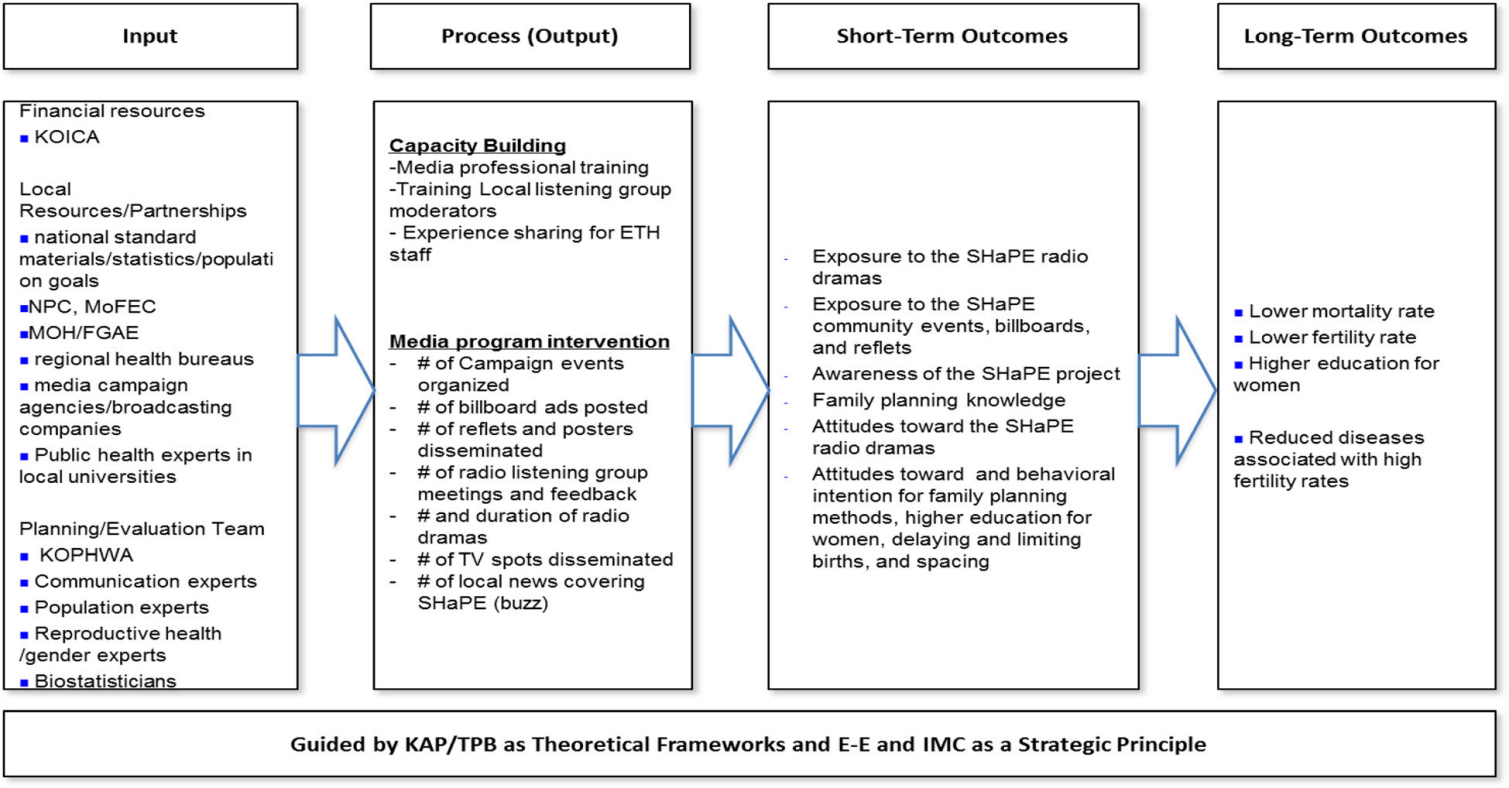
The SHaPE logic model

Alongside its multi-media campaign, SHaPE includes two other components: (1) capacity building by training public health and media staffs and professionals; and (2) advocacy activities to build support that may affect policies. In this paper, though, we focus only on the multimedia campaign protocol, divided into the following three components: (1) a nationwide representative survey serving as formative research to identify region-specific and culturally appropriate media, messages, and determinants of family planning; (2) the multimedia campaign intervention; (3) campaign evaluation, including pre-, process-, and post-evaluation research using both quantitative and qualitative methodologies. The logic model of the SHaPE campaign is presented in Fig. 1.

### Theoretical framework

SHaPE is guided by the two theoretical frameworks of Knowledge, Attitudes, and Behavior (KAB), as often known as Knowledge-Attitude-Practice (KAP), and the Theory of Planned Behavior (TPB), as well as strategic tools such as Entertainment Education (E-E) and Integrated Marketing Communications (IMC). KAB explains that behavior change is followed by gradual and step-by-step changes of knowledge and attitudes [24]. In FP literature, lack of information has been found to cause unwanted pregnancy and unintended childbearing, and KAB has usefully highlighted the importance of providing information in this context [12]. The basic idea is that providing information and improving knowledge could result in behavior changes. KAB has also provided theoretical justifications for information campaigns [25].

But since knowledge alone is not a sufficient prerequisite for behavior change, TPB supplements KAB with the additional components of subjective norms and perceived behavioral control. The TPB assumes that behavior depends on intention, which is determined by a person’s attitudes, subjective norms, and perceived behavioral control [26]. Here, *attitudes* refer to evaluative judgments toward a desired or recommended behavior. *Subjective norms* refer to people’s beliefs about how their significant others will regard them if they do or do not adopt a behavior. Last, *perceived behavioral control* refers to people’s perceptions of their ability to perform the behavior. Perceived behavioral control is equivalent to a concept from Bandura’s social cognitive theory, *perceived self-efficacy*, which refers to people’s judgments of how well they themselves can execute courses of action required to deal with prospective situations [27, 28]. Subjective norms are particularly important in our project settings, where religious norms, gender norms, and cultures influence people’s family planning behaviors [29]. The concepts that constitute KAB and TPB were operationalized for use in baseline surveys, as well and pre- and post-evaluation surveys. In addition, these concepts were taken into consideration during the development of key concepts and messages for the radio dramas and other media campaign materials.

Turning to strategic tools, E-E is a communication strategy that inserts educational or motivational information into entertainment media programs, for example dramas [30]. Because entertainment programs have narratives or stories with intricate plots and interesting characters, audiences can become involved with the narratives and often identify with the main characters. E-E is effective in behavior changes because, according to social learning theory [31, 32], people tend to adopt a behavior faster if they are motivated by those they consider to be role models. E-E has been popular because it affords opportunities to reach a large audience and to address issues that are sensitive or culturally taboo in an entertaining and informative manner [33]. A meta-analytic study of 22 published studies on the effects of E-E found that the strategy had significant effects on knowledge, attitudes, behavioral intention, and behaviors [34].

In Ethiopia, a specialized form of E-E, called the Sabido method, was employed to produce a radio serial drama on issues of family planning, HIV/AIDS, and reproductive health [35]. The Sabido method is unique because it mainly uses the serial drama format for conveying prosocial messages, and it constructs characters as “vicarious role models” intended to provoke positive changes [36]. Because radio was Ethiopia’s most widely accessed media channel, SHaPE employed this method to produce radio soap dramas that are culturally appropriate to each of the intervention sites.

Empirical evidence has found E-E to be most effective when combined with other intervention components such as interpersonal communication [33]. Accordingly, SHaPE incorporated other media and interpersonal/community-level media. In advertising and marketing research, the integration of different media channels and marketing communication techniques is known as Integrated Marketing Communications (IMC). The purpose of IMC is to deliver unifying messages in a consistent manner through multiple channels in order to maximize campaign effectiveness. IMC has informed not only marketing communication but also communication for public and other health issues [37, 38].

### Setting and participants

The main target population for the SHaPE multimedia campaign is reproductive age women and men in the three regions -- Amhara, Oromia, and Somali – which take up about 66.6% of the entire country and have distinct ethnicities, cultures, and languages [10].

### SHaPE components

#### National-level survey

##### Purpose

The national survey served as formative research, the purpose of which was to identify determinants of family planning, barriers against and benefits of FP, media use, specific cultural values and norms relevant to FP, unmet needs, and barriers against FP uptake, particularly in the three target regions. Survey findings were used to determine what should be the campaign’s key messages, what other types of media should be used together with radio dramas, and whether there are any community issues and concerns that the media campaign should address.

##### Sampling

The data were collected between December 2016 and January 2017 using two-stage stratified cluster sampling methods. First, 30 zones were selected using simple random sampling from five regions -- Amhara, Oromia, SNNPR, Tigray, and Somali – as well as from the Addis Ababa city administration. Next, districts were stratified according to place of residence as rural and urban within their respective selected zone. The proposed 3 districts were then allocated proportionally according to their weight to each stratum (urban, rural) in each of the 30 zones. The required 69 districts were selected using simple random sampling from both the urban and rural districts. Then, a total of 207 (3 from each district/sub-city) enumeration areas, taken from the national central statistics agency, were selected. The total sample size (11,969 households) was allocated based on probabilities proportional with size to each enumeration area within each district (stratum). Last, households were selected by proximate sampling from each enumeration area, with 30–50 interviews conducted per area. The overall response rate was 95%, with 7696 women and 3669 men.

##### Survey instrument

The survey data were collected by trained data collectors, using the Research Electronic Data Capture (REDCapAP) software and data collection tool installed on smartphones. Two questionnaires were used, one for men and the other for women. The study target for women was ages 15–49. The women’s questionnaire included questions on the following topics: background characteristics (including age, education, and media exposure); birth history and childhood mortality; and family planning questions such as knowledge, contraceptive use, beliefs/norms/attitudes, sources of contraceptive methods, fertility preferences, women’s work and husbands’ background characteristics, and media related questions. The study target for men was ages 15–59. Their questionnaire was similar to the women’s but excluded questions related to reproductive history that only women can answer.

#### Multimedia campaign

The SHaPE multimedia campaign included radio dramas as the main component, along with TV and radio spots, community events/road shows, print media such as brochures/leaflets and posters, and billboards. Radio dramas served as the key campaign component to increase FP-related knowledge, attitudes, and behavior by providing entertaining narratives on male and female role models. Other supplemental media were used to inform and educate specific family planning services and to advertise community events. The following steps were taken to design and implement the campaign (see Table 1 for campaign activities).

- Step 1 (agency selection): A SHaPE multimedia campaign agency was selected through an open and competitive bidding procedure nationwide in Ethiopia.
- Step 2 (concept development): The formative survey findings and FP literature reviews were used to develop evidence-based key concepts and messages for the radio dramas and other media channels to be integrated per region.
- Step 3 (partnership building/finalizing): Partnerships were established with key federal and regional/local stakeholders to implement the campaign.
- Step 4 (production): Communication strategies were finalized, and creative brief and draft contents were developed for a radio serial drama and other media campaign activities/materials.
- Step 5 (pretest): The campaign materials were pretested to ensure feasibility and effectiveness of the messages, design, and media strategy. All the materials were written in Amharic, the official language, and then translated into the other two local languages, Oromiffa and Somali.
- Step 6 (launching): The multimedia campaign was launched with a launching event and media briefing both at the federal level and in each respective region.
- Step 7 (implementation): The multimedia campaign was implemented in Amhara, Oromia, and Ethiopian Somali regional states from February 2017 to February 2018. Promotional materials such as T-Shirts, caps, stickers, and stretch and roll-up banners were distributed at mass gathering events organized in major towns of the three target regions for IMC.

**Table 1:** Campaign activities

	Oromia	Amhara	Ethiopia Somali
RADIO			
Name of the drama	Abdi boruu	Yedegu lijoch	Dhaqasho
# of episodes (minutes)	35 (20) + 1 rerun	35 (20)	35 (20)
Language	Afan Oromo	Amharic	Somali
Period	9/6/2017 ~ 9/2/2018	4/7/2017 ~ 19/12/2017	28/8/2017 ~ 25/12/2017
Storyline (or key concept)	An extended family struggling to have a better life for their children future by sending the girls to school and by using FP methods properly.	A family who learns about the benefit of family planning based on the assumption that increased number of children causes scarcity of resource and unstable life.	How FP/ child spacing helps the mother to have time to beautify herself to her husband so that he does not go to another woman, proofing how child spacing helps a mother bring up an educated, healthy and wealthy child, confirming the opportunity child spacing gives a mother to empower herself and having happy family.
TV Spots (frequency)	4 (40)	4 (40)	4 (40)
Radio Spots (frequency)	4 (120)	4 (120)	4 (120)
Areas locating Campaign Billboards (N)	Adama (2)	Bahirdar (2)	Jigjiga (2)
	Assela (1)	Debrebrihan (1)	Degehabur (1)
	Chiro (1)	Desse (1)	Kebridehar (1)
	Jimma (1)	Debere markos (1)	Togwochale (1)
Posters	5000	5000	12,000
Booklet	5000	5000	5000
Leaflet	5000	5000	5000
DVD	1000	1000	
# of areas for road/screen show	20 towns	20 towns	20 towns

### Evaluation methods

#### Monitoring/process research: Radio listening group

In each region, a radio listening group was established for monitoring and process-evaluating the radio drama as the main component of the campaign. These listening groups were used during the campaign implementation to determine dose and fidelity [39], to check for any technical difficulties in the dramas’ radio transmission, and to get audience feedback about the dramas.

The Family Guidance Association of Ethiopia (FGAE), which partnered with SHaPE, used its nationwide networks to recruit 750 participants (250 from each region) and 20 facilitators, and to hold meetings in its regional offices. The FGAE’s regional and district coordinators trained the facilitators, held meetings at 10 operating sites, and managed the quality of the 75 bi-weekly listening groups. There were 10 participants per group, and each person was provided with a radio device.

In each meeting, a facilitator asked the participants to fill out a checklist and then moderated focus group discussions. The checklist, which served as a quantitative assessment tool, included a short list of questions with simple “yes, no, or don’t know” response options. The questions covered the following three issues: (1) Access to the radio drama (e.g., How many episodes were transmitted for the last two weeks? Was the radio drama transmitted on schedule? Was it transmitted clearly?); (2) understanding of the drama (e.g., Was it easy to understand, interesting, memorable, boring, culturally appropriate, likeable?); and (3) effectiveness of the drama (Did you like the actor, recall the main message, find the message important, intend to change your behavior, or intend to discuss the topic with others?). In addition, a standard focus group moderator’s guide was used to facilitate in-depth discussions of the participants’ reactions to the drama. In the first meeting, additional socio-demographic questions were asked to capture how each participant might change over time with respect to understanding, attitudes, and behaviors. To get timely feedback for subsequent radio drama episodes, the media campaign agency received the qualitative discussion feedback after each focus group session.

## Pre- and post-media campaign assessment survey

To assess the campaign effectiveness, pre- and post-campaign evaluation research was conducted using both quantitative and qualitative methods.

### Quantitative assessment

**Fig. 2:**
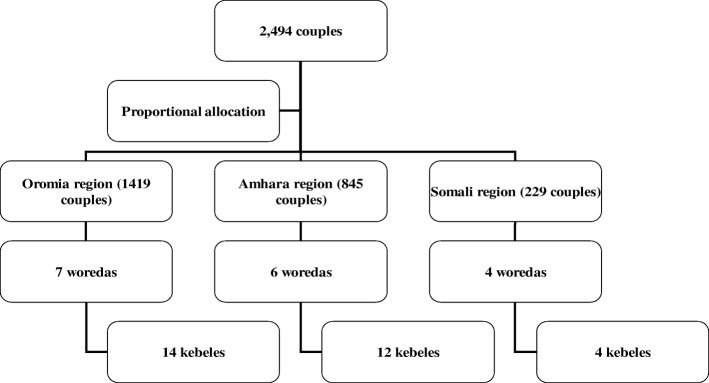
Schematic presentation of the sampling procedure

A multilevel and multistage cluster sampling method was used to identify study participants, 2494 couples living in closely located households in Gott. Gott usually consisted of between 150 and 200 homogenous households, which are nested in sections called Kebeles, which themselves are nested in districts called Woredas. The calculated total sample was distributed among the three regions proportionally using the projected number of reproductive age women for the year 2017 in each study region (Table 2). The estimated size of the population from the study’s Kebeles and Woredas was received from Woreda health offices. This sample was proportionally allocated, and three clusters (EAs) from each Kebele were selected. The study participants were proportionally allocated to urban and rural Kebeles. In the post-campaign study, the same clusters (EAs) selected in the base line were followed, and randomly selected households from the clusters were surveyed (see Fig. 2 for schematic presentation of the sampling procedure). This is a repeated, cross-sectional, cluster-randomized design, which has been used to measure campaign effectiveness [40], particularly when a panel design is ideal for tracking individual changes in knowledge, attitudes, and behaviors. However, this design is not realistic at a large scale.

**Table 2:** The distribution of study couples by study region

Region	Projected Population in 2017	Women of reproductive health ages in 2017	Percent	Study Couples
Amhara	21,134,988	4,966,722	33.9	845
Oromia	35,467,001	8,334,745	56.9	1419
Somali	5,748,998	1,351,015	9.2	229
Total	62,350,987	14,652,482	100	2494

The face-to-face survey was conducted by trained interviewers from the respective regions. For the sake of gender sensitivity, two trained male and female interviewers visited each household to survey male and female participants respectively. If a person was not at home, appointments were made to administer the survey on a subsequent visit. Tablets in which an Open Data Kit (ODK) application was installed were for data collection to minimize errors by developing a data entry template with an internal consistency check. This procedure also helped minimize the data entry time.

The survey instrument comprised six components for both pre- and post-survey: (1) study identification (e.g., study region, name of village, date, time, and duration of the interview); (2) social-demographic and economic characteristics of the respondents (e.g., age, sex, ethnic group, religion, residence, household size); (3) access to media channels (radio/print/TV/Internet media use and frequency); (4) reproductive health questions (e.g., age of first pregnancy, number of children, breastfeeding, knowledge and use of family planning (FP) methods, sources of information about FP, interpersonal communication about FP, social support, social norms, attitudes); (5) gender and self-efficacy-related questions (e.g., Men should have a higher educational level than women. Only women are responsible for household chores); and (6) knowledge, subjective norms, and attitudes regarding FP. The campaign-related questions were included only for post-survey, which include awareness of SHaPE and campaign messages, exposure to campaign materials, recall of the messages, attitudes, and behavioral intention.

### Qualitative assessment

A case study method and a key informant interview were conducted before and after the campaign. For the case study, interviews were conducted among a total of 24 cases/couples -- 8 in each region including modern family planning users and non-users. The cases in each region were a mix of women and men, including 4 current modern FP users and 4 non-users.

A total of 72 key informant interviews (24 per region) were conducted with the following composition specially arranged: two religious leaders from each study (one Muslim and one Christian), a family planning provider (professional health personnel), and a program leader. Either a trained health extension worker or a member of the Women’s Development Army interviewed the case couples and key informants from each of the study districts.

The qualitative assessment tools included questions about the following topics: (1) modern FP methods and counseling services available in a particular health institution; (2) FP services available in the nearby health institutions, including short- or long-term methods depending on demand; (3) currently used FP methods; (4) reasons for not using FP methods; (5) male involvement in FP; (6) partners who support FP services or interventions, and types of support from the partners (only for health workers); (7) decision for doing FP; and (10) beliefs about and challenges for FP.

## Discussion

The Small, Happy, and Prosperous Family in Ethiopia (SHaPE) is one of Ethiopia’s first comprehensive multimedia campaigns focusing on family planning and covering the nation’s major regions. It combines radio drama with other forms of interpersonal, community-level, and mass media communication. Its purpose is to enhance the FP-related knowledge, attitudes, and practices of Ethiopians, particularly women of reproductive age. It contributes to existing family planning research and intervention because it is theory- and evidence-based, and because it employs integrated marketing communications and entertainment-education approaches with key messages that have been tailored for culturally specific audiences. Alongside the media campaign, SHaPE also incorporates capacity building and advocacy to build a sustainable FP infrastructure for Ethiopia. However, it should be noted that, even within the same country, a nationwide campaign with uniform messages is neither possible nor desirable when there are different cultures, norms, and languages across regions. Also, media campaigns in developing and underdeveloped countries need to incorporate constant monitoring of political situations because political instability often hinders the ability to conduct a campaign in a timely and consistent manner. Nevertheless, carefully designed and implemented campaigns and evaluation research can provide lessons for future FP campaigns, not only in Ethiopia but in other developing and underdeveloped countries.
